# Complete Genome Sequence of Hemolysin-Containing *Carnobacterium* sp. Strain CP1 Isolated from the Antarctic

**DOI:** 10.1128/genomeA.00690-16

**Published:** 2016-07-21

**Authors:** Sidong Zhu, Xing Wang, Di Zhang, Xiaohuan Jing, Ning Zhang, Jifang Yang, Jigang Chen

**Affiliations:** College of Biological and Environmental Sciences, Zhejiang Wanli University, Ningbo, Zhejiang, China

## Abstract

*Carnobacterium* sp. strain CP1 was isolated from Antarctic sandy soil and predicted to be a novel species belonging to the genus *Carnobacterium*. Herein, we report the complete genome sequence, which consists of a circular 2,605,518-bp chromosome and an 8,883-bp plasmid with G+C contents of 38.13% and 31.63%, respectively.

## GENOME ANNOUNCEMENT

*Carnobacterium* sp. strain CP1 was isolated from sandy soil in David Station (S68°34.75′, E77°57.75′) in eastern Antarctica. The 16S rRNA gene sequence revealed 97.6% similarity with *Carnobacterium mobile* DSM 4848^T^ (1). Based upon cladistic and phenetic analyses, this strain was predicted to be a novel species belonging to the genus *Carnobacterium* (Zhu S, Wang X, Xiong S, Wang P, Wang H, Yang J, Chen J, unpublished data). Herein, we report the complete genome sequence that encodes a beta-hemolysin.

*Carnobacterium* sp. CP1 was cultured in tryptic soy broth (TSB) medium, and genomic DNA was isolated using the cetyltrimethylammonium bromide (CTAB)-NaCl method, as previously described (2). The complete genome was sequenced by Nextomics Biosciences Co., Ltd. (Wuhan, China) using a single-molecule real-time (SMRT) sequencing approach. Genomic DNA was interrupted randomly and repaired to construct a DNA library, which was then qualified using an Agilent 2100 Bioanalyzer high-sensitivity kit. DNA fragments were sequenced using a PacBio RS II SMRT DNA sequencing system (3, 4). Finally, reads were assembled using the Hierarchical Genome Assembly Process 2.2.0 software (4). Two SMRT cells were sequenced, and 1.05 Gb of data were obtained. Reads between ~10 bp and 20,000 bp were preassembled using Basic Local Alignment with Successive Refinement (BLASR) (5) and aligned using the Celera Assembler (6). Annotation was performed using the combined results from RAST (7) and Glimmer version 3.0 (8). tRNAs and rRNAs were identified using the tRNAscan-SE (9), RNAmmer (10), and Rfam (11) databases.

The complete genome of *Carnobacterium* sp. CP1 consists of a circular 2,605,518-bp chromosome and an 8,883-bp plasmid with G+C contents of 38.13% and 31.63%, respectively. The chromosome contains 2,404 predicted protein-coding genes (CDSs), of which 79.86% were annotated as proteins with known biological function, and 28.61% were annotated as hypothetical proteins. Additionally, 77 tRNAs, eight large rRNA subunits, eight small rRNA subunits, and nine 5S rRNAs were identified. The plasmid contains 13 predicted CDSs, of which six were annotated as proteins of known function and seven were hypothetical proteins. The RAST server also revealed the presence of 341 subsystems based on annotation of the whole genome.

The genome of CP1 includes an extensive set of stress response genes (74 in total), which is an indication of its genetic adaptation to the extreme environmental conditions (hyperaridity, strong UV radiation, and cold temperatures) encountered in Antarctica. A number of genes linked to biotic and abiotic defense mechanisms (46 in total) were also detected. The genome displays high metabolic versatility, with 349 genes related to carbohydrate metabolism, and 36 and seven genes associated with phosphorous and sulfur metabolism, respectively. The genome also encodes numerous proteins for the biosynthesis, degradation, and metabolism of amino acids, with 32 annotated genes directly linked to the biosynthesis of aromatic amino acids and their derivatives alone. A remarkable feature of the CP1 genome is the inclusion of four genes linked to secondary metabolism, specifically, auxin biosynthesis, and a further two genes encoding the membrane protein hemolysin III. However, genes encoding toxins, virulence factors, pathogenicity islands, and transposable elements are not present in the CP1 genome.

### Nucleotide sequence accession numbers.

The complete genome sequence of *Carnobacterium* sp. CP1 has been deposited in GenBank with the accession numbers CP010796 (chromosome), and CP010819 (plasmid pCP1).
